# Current Status Regarding Immunosuppressive Treatment in Patients after Renal Transplantation

**DOI:** 10.3390/ijms241210301

**Published:** 2023-06-18

**Authors:** Kamila Szumilas, Aleksandra Wilk, Piotr Wiśniewski, Anna Gimpel, Violetta Dziedziejko, Markus Kipp, Andrzej Pawlik

**Affiliations:** 1 Department of Physiology, Pomeranian Medical University in Szczecin, 70-111 Szczecin, Poland; kamila.szumilas@pum.edu.pl; 2 Department of Histology and Embryology, Pomeranian Medical University, 70-111 Szczecin, Poland; aleksandra.wilk@pum.edu.pl (A.W.); aniagimpel@gmail.com (A.G.); 3 Department of Nephrology, Transplantology and Internal Medicine, Pomeranian Medical University, 70-111 Szczecin, Poland; piotr_wi@yahoo.pl; 4 Department of Biochemistry and Medical Chemistry, Pomeranian Medical University, 70-111 Szczecin, Poland; viola@pum.edu.pl; 5 Institute of Anatomy, Rostock University Medical Center, Gertrudenstrasse 9, 18057 Rostock, Germany; markus.kipp@med.uni-rostock.de

**Keywords:** renal transplantation, immunosuppressive treatment, immunosuppressive drugs

## Abstract

Renal transplantation is now the best treatment for end-stage renal failure. To avoid rejection and prolong graft function, organ recipients need immunosuppressive therapy. The immunosuppressive drugs used depends on many factors, including time since transplantation (induction or maintenance), aetiology of the disease, and/or condition of the graft. Immunosuppressive treatment needs to be personalised, and hospitals and clinics have differing protocols and preparations depending on experience. Renal transplant recipient maintenance treatment is mostly based on triple-drug therapy containing calcineurin inhibitors, corticosteroids, and antiproliferative drugs. In addition to the desired effect, the use of immunosuppressive drugs carries risks of certain side effects. Therefore, new immunosuppressive drugs and immunosuppressive protocols are being sought that exert fewer side effects, which could maximise efficacy and reduce toxicity and, in this way, reduce both morbidity and mortality, as well as increase opportunities to modify individual immunosuppression for renal recipients of all ages. The aim of the current review is to describe the classes of immunosuppressive drugs and their mode of action, which are divided by induction and maintenance treatment. An additional aspect of the current review is a description of immune system activity modulation by the drugs used in renal transplant recipients. Complications associated with the use of immunosuppressive drugs and other immunosuppressive treatment options used in kidney transplant recipients have also been described.

## 1. Introduction

Renal transplantation is now the best treatment for end-stage renal failure. An important issue in renal transplantation is the fact that immunosuppressive drugs exert many side effects, including nephrotoxicity. It seems problematic that on one hand, they protect transplanted kidneys, while on the other hand, they negatively affect kidney function [1].

The gold standard immunosuppressive therapy for renal transplantation uses multiple agents with different targets and mechanisms of action: (1) the calcineurin inhibitors (CNIs) (cyclosporine and tacrolimus), (2) mammalian target of rapamycin inhibitors (sirolimus and everolimus), (3) antiproliferatives (azathioprine and mycophenolic acid derivatives), (4) glucocorticosteroids, and (5) biological immunosuppressive agents. It should be emphasised that the treatment for patients who have undergone organ transplantation needs to be individualised [2,3,4].

**Figure 1:**
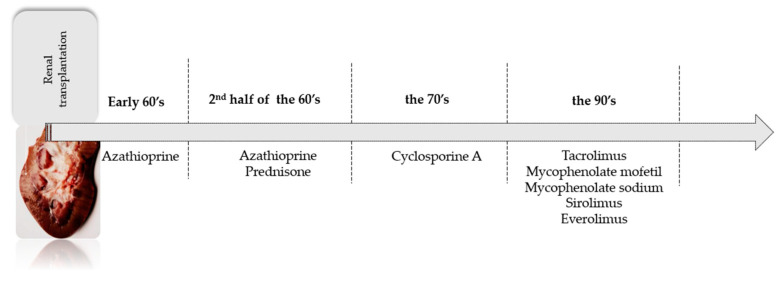
The inclusive history of individual immunosuppressive drugs.

Today, new generations of immunosuppressive drugs are still needed, with the aim of prolonging graft function. Trends have changed over time, and the inclusive history of individual immunosuppressive drugs is shown in Figure 1. Recently, glucocorticosteroids were found as not always necessary in maintenance therapy. Moreover, new generation drugs make more individualised treatment possible. The selection of an appropriate immunosuppressive regimen should be patient-specific, considering the medications’ pharmacologic properties, adverse event profile, and potential drug–drug interactions, as well as the patient’s pre-existing diseases, risk of rejection, and existing medication regimen.

The aim of the current review is to describe the classes of immunosuppressive drugs and their mode of action with division on induction and maintenance treatment. The aim of the current review is to describe the classes of immunosuppressive drugs and their mode of action divided by induction and maintenance treatment. An additional aspect of the current review is a description of immune system activity modulation by the drugs used in renal transplant recipients.

## 2. Immunological Aspects of Transplantation

Transplantation refers to the transfer of tissue or an organ to the same person (autogenous/autologous) or another person (allogeneic). In addition to strictly regulated legal requirements, in the case of allotransplantation, a high degree of histocompatibility between the donor and recipient is usually desired to reduce the risk of organ rejection. Since a complete match of the major histocompatibility complex (MHC) can only be realised in isograft transplantation (the transfer of genetically identical material, such as between identical twins), immunosuppressive therapy is generally required following tissue or organ transplantation. Of note, there are some exceptions, such as corneal transplants which are rarely rejected because of the lack of corneal blood supply. This chapter provides an overview of solid organ transplantation (in this case, the kidney) and the immunological challenges that come with such a procedure.

The transplantation of an antigenically foreign tissue or organ is associated with the activation of a cascade of immune mechanisms in the recipient aimed at rejecting it [5,6]. When a foreign organ, such as a kidney, is transplanted into a non-identical individual of the same species, the organ is called an allogeneic transplant. The immune response from the recipient to the allograft is called the alloimmune response, which is initiated by the recognition of alloantigens by T cells (commonly known as allorecognition). Allorecognition is the first step in a series of complex events that lead to T-cell activation (i.e., cellular immunity), antibody production (i.e., humoural immunity), and allogeneic graft rejection [7,8,9]. Of note, alloreactive T cells are readily detectable in naive animals and humans at surprisingly high frequencies [10]. Mismatched or inadequately matched organs can trigger transplant rejection, which can manifest in three forms: hyperacute rejection, acute rejection, and chronic rejection.

Hyperacute rejection occurs within minutes to hours after transplantation and is caused by pre-existing donor-specific antibodies in the recipient that recognise antigens in the transplanted kidney. The presence of those antibodies is due to prior sensitisation of the patient from blood transfusions, pregnancy, or a previous transplantation. This type of rejection can cause irreversible damage to the graft, leading to graft necrosis and failure. Hyperacute rejection is extremely rare today due to the universal adoption of pretransplant crossmatching techniques.

Acute rejection can occur within the first week to three months after transplantation and can arise from different immunological mechanisms. Acute cellular rejection occurs due to cytotoxic T cells that attack the transplanted tissue. Distinct mechanisms might play a role. Firstly, the dendritic cells (DCs) can migrate from the transplanted kidney to lymphoid tissues of the recipient and present their foreign MHC peptides to the recipient lymphocytes. Alternatively, dendritic cells of the recipient present, after internalisation and processing, peptides from the donor tissue. The first scenario is called the direct, whereas the latter one is called the indirect, allorecognition pathway [6,7]. In consequence, an acute cellular rejection occurs by cytotoxic T cells that begin to secrete cytokines to recruit more lymphocytes as well as cause apoptosis or cell death. Acute humoral rejection is initiated by helper T cells in the recipient, leading to the creation of donor-specific antibodies that deposit within the donor graft and activate the complement cascade and antibody-mediated cytotoxicity.

Chronic rejection is an insidious form of rejection that leads to graft destruction over months or years after transplantation. Several different factors contribute to this form of rejection including a persistent allogeneic immune response leading to vascular or parenchymal damage and, finally, organ fibrosis. Beyond, non-immunological factors might play a role, such as nephrotoxicity due to long-term exposure to calcineurin inhibitors (e.g., cyclosporine or tacrolimus that can produce interstitial fibrosis) [11] as well as viral infections or cancer due to the treatment-induced immunosuppression [12]. Unfortunately, there is currently no cure for chronic rejection other than removing the graft.

Occasionally, a patient may encounter a phenomenon referred to as “graft versus host reaction,” in which immune cells already present in the donor graft start to attack the recipient’s healthy cells. Graft versus host reaction is a significant concern with stem cell transplants, including bone marrow transplants, and it can also arise following blood transfusions when the donor graft is considered “immune-competent” or capable of initiating an immune response. Of note, graft versus host disease is a rare complication after kidney transplantation [13,14].

There is some debate regarding the direct pathway of allorecognition. As outlined, in this scenario, allograft rejection is triggered by the migration of donor-derived dendritic cells from the transplanted kidney into the recipient’s lymph nodes (or other secondary lymphatic tissues). Within the lymphatic tissue, donor-derived dendritic cells present donor-derived peptide–MHC complexes directly to recipient T cells, which is followed by T-cell activation. Several observations support this theory: for example, leukocyte-depleted grafts were not rejected in murine models [15], and donor-derived DCs were present in recipient lymph nodes in a mouse cardiac allograft model [16]. In line with this assumption, depleting those “travelling” dendritic cells (named passenger leukocytes in a recent review from Duneton and colleagues [17]) significantly prolonged the survival of heart allografts [18]. In contrast to these findings, the depletion of passenger leukocytes had limited impact on kidney graft survival in a porcine model [19]. In support of the indirect pathway of allorecognition, later studies have reported that donor-derived DCs in grafts decrease over time and are replaced by recipient DCs. This finding suggests that alloantigens may also be recognised conventionally, meaning that recipient antigen-presenting cells process and present donor self-peptides (i.e., indirect pathway) [17]. Many studies have shown this having a pivotal role during graft rejection. Of note, the result of a clinical study suggests that the indirect pathway of allorecognition is mainly responsible for chronic allograft nephropathy [20], whereas the direct pathway of allorecognition appears to be more important for acute rejections, which coincides with the limited lifespan of donor-derived DCs.

After the antigen presentation processes via the direct and indirect pathway occur, naive alloreactive T cells are primed in recipient lymph nodes, and CD4+ and CD8+ T cells differentiate into effector cells, which play important roles in graft rejection. CD4+ cells differentiate into various subsets such as TH1, TH2, TH17, T follicular helper, and regulatory T (Treg) cells, depending on the cytokine microenvironment. These cells can perform various functions, including direct cytotoxicity, cytokine secretion, and the provision of assistance to cytotoxic CD8+ T cells and B cells, the latter for producing alloantibodies. CD8+ T cells can directly eliminate cells that present non-self-epitopes by releasing cytotoxic molecules (e.g., granzymes and perforin) or inducing apoptosis through cell surface interactions (e.g., binding of FAS ligand (CD95L) on T cells to FAS on target cells).

Activated cognate CD4+ and CD8+ T-cell populations infiltrate the graft and orchestrate an inflammatory response within the kidney interstitium by engaging APCs. In principle, the activation, differentiation, and expansion of CD4+ and CD8+ T cells are dependent on three signals: antigen recognition via T-cell receptor (TCR)–MHC interactions (signal 1), co-stimulation, including CD28-CD80/CD86 on antigen-presenting cells (APCs) or CD40–CD40L (CD154) interactions (signal 2), and cytokine-mediated signals (signal 3). Of note, the local innate immune system, which consists of macrophages, neutrophils, dendritic cells, and natural killer cells, can shape the activation of CD4+ and CD8+ cells via the secretion of pro-inflammatory cytokines when it occurs in the transplanted kidney [21]. On the histopathological level, this inflammatory response leads to the loss of parenchymal cell function and intimal arteritis.

**Figure 2:**
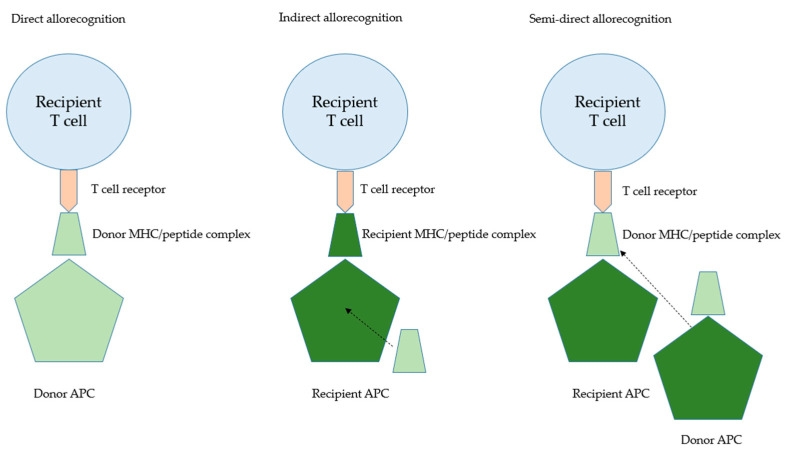
T-cell recognition pathways.

**Figure 3:**
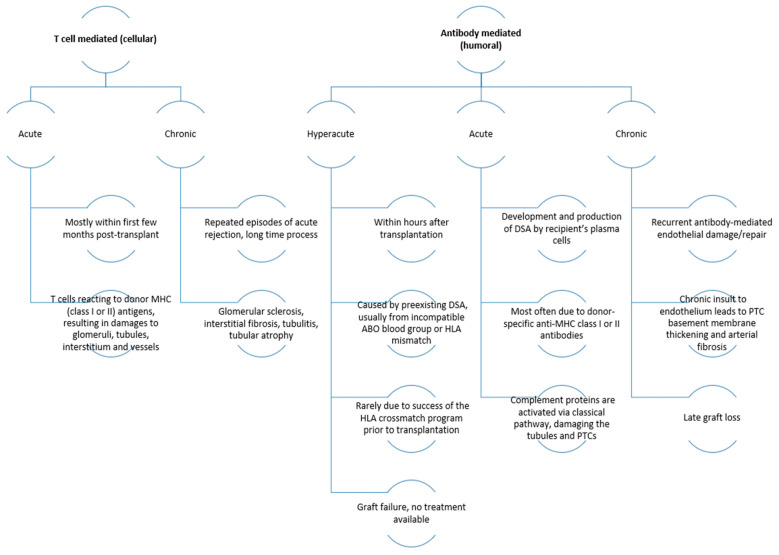
The types of rejection with their characteristics [22]. Abbreviations: DSA, donor-specific antigen; HLA, human leukocyte antigen; MHC, major histocompatibility complex; PTC, peritubular capillary.

Finally, in this section, we would stress that the transplanted kidney is by no means a healthy organ but represents an organ with a significant ischaemia–reperfusion injury. During transplantation, ischaemia–reperfusion injury occurs when blood supply is suddenly restored to the graft after a period of interrupted perfusion, leading to sterile inflammation. This inflammation results in cell death and tissue damage, releasing endogenous damage-associated molecular patterns (DAMPs) that are typically concealed from the immune system within cells and act as “danger signals”. Pattern recognition receptors (PRRs), such as toll-like receptors (TLRs), recognise these DAMPs, and the innate immune system responds accordingly. Due to intimate interactions of the adaptive and innate immune system, ischaemia–reperfusion injury-triggered innate immune activation can also impact adaptive pathways implicated in acute and chronic graft rejection. Allorecognition pathways are additionally presented in Figure 2, and the types of rejection with their characteristics are presented in Figure 3.

**Figure 4:**
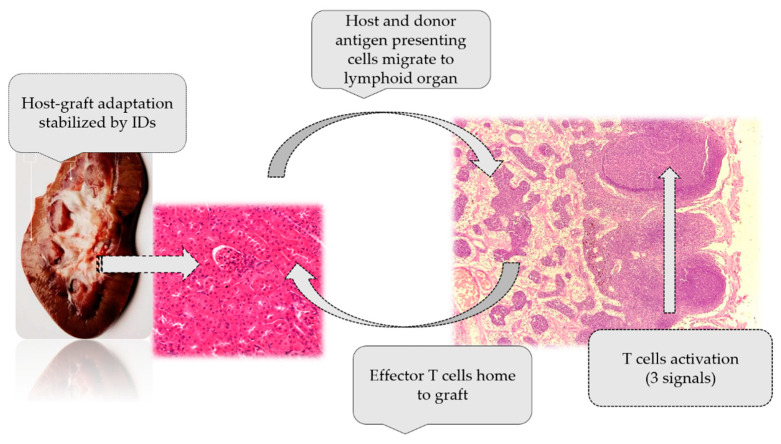
Mechanism of action of immunosuppressive drugs [1].

Additionally, mechanism of action of immunosuppressive drugs was presented in Figure 4.

## 3. Classes of Immunosuppressive Drugs

**Figure 5:**
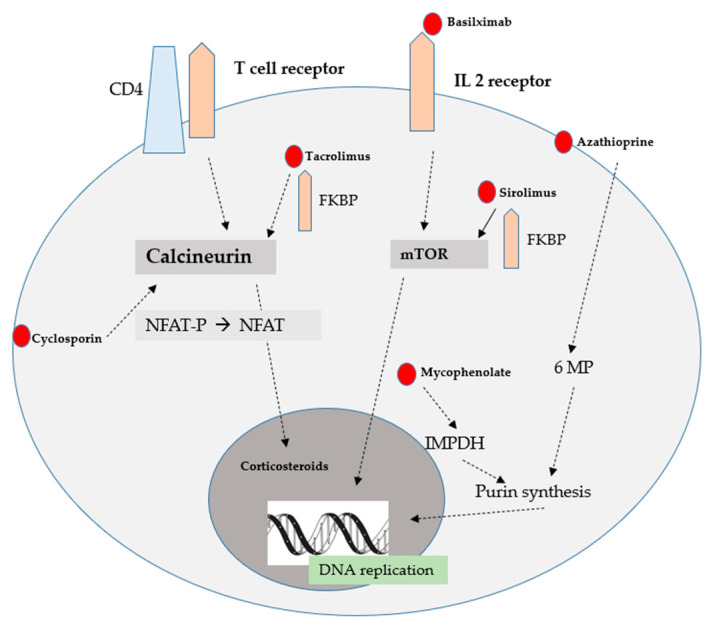
The mode of action of immunosuppressive drugs [1].

There are five classes of the most used immunosuppressive drugs in renal transplant recipients. Additionally, the mode of action of immunosuppressive drugs is presented in Figure 5.

### 3.1. Immunosuppressive Drugs—Induction Therapy

Biological agents, such as antibodies, have been developed for use in induction therapy or in the treatment of transplant rejection. They regulate the immune response in various ways. Induction drugs can inhibit lymphocytes or prevent their activation and replication, such as the IL-2 receptor antagonist (IL-2RA) [23].

The main induction drugs in kidney transplantation are basiliximab, alemtuzumab and antithymocyte globulin [24].

Basiliximab is a chimeric monoclonal antibody directed against the alpha chain of the interleukin-2 receptor (IL-2R). It is involved in binding to and blocking this chain, thereby preventing IL-2R activation.

Alemtuzumab promotes the lysis of T and B lymphocytes, monocytes and NK cells in the peripheral blood, leading to the profound and prolonged depletion of T lymphocytes and more transient depletion of B lymphocytes and monocytes [25].

In addition to monoclonal antibodies, polyclonal antibodies are commonly used by organ recipients.

One of the mentioned antibodies is anti-thymocyte globulin. These antibodies are produced by immunising animals with human lymphoid cells. The most suitable antibodies recognise the markers CD2, CD3, CD4, CD8, CD11a, CD18, CD25, CD28, CD40 and CD54 and have a broad immunosuppressive spectrum compared to monoclonal antibodies [25]. Hafeez et al. [26] examined the anti-thymocyte globulin aspect of induction to deceased donor kidney transplants. The authors indicated favourable rejection, patient survival and graft survival with anti-thymocyte globulin usage [26]. It should be added that Muromonab-CD3, often referred to as OKT3, was previously used to treat acute cellular rejection after solid organ transplantation. However, due to hepatotoxic properties, the mentioned drug was withdrawn from use in 2010 [27,28,29,30].

Other biologic drugs, such as eculizumab, belatacept, and rituximab, are often used in specific clinical conditions [31].

Eculizumab is monoclonal antibody that recognises the C5 complement chain protein, inhibiting its degradation into C5a and C5b. By blocking the formation of C5b, it inhibits the subsequent formation of the membrane-attacking complex, C5b-9 or MAC [25]. It can be successfully used in patients with atypical haemolytic uremic syndrome (aHUS) [32]. Siedlecki et al. [33] indicated that the outcomes of transplant patients with aHUS treated with eculizumab were better compared to previous reports of aHUS patients not treated with eculizumab [33].

Another protein, belatacept, also used in the prevention of acute cellular rejection, contains the CTLA-4 molecule fused to the Fc domain of human IgG1 [25,31]. This protein binds to the CD80 and CD86 molecules on APCs and prevents their interaction with the CD28 molecule of T lymphocytes, blocking co-stimulation, the second signal of lymphocyte activation; thus, belatacept is a selective blocker of T lymphocyte co-stimulation [25]. According to Lombardi et al. [34], this drug may increase the risk of graft rejection, especially within the first year after start of treatment. Data also indicate this drug may increase risk for CMV-disease. Regarding the viral aspect, belatacept reduces the response rate to the anti-SARS-CoV2 mRNA-based vaccination. Due to scarce data based on renal transplant recipients receiving belatacept, most questions regarding advantages and disadvantages remain without answer [34]. Belatacept is only used in a few countries, but it is the only immunosuppressive drug administered intravenously monthly [35].

Rituximab is an antibody that blocks B cells and prevents or delays relapse in patients suffering from nephrotic syndrome [36]. It can be used in patients with focal segmental glomerular sclerosis (FSGS), although results are inconclusive [37,38,39].

### 3.2. Immunosuppressive Drugs—Maintenance Therapy

#### CNIs

There are two drugs belonging to calcineurine inhibitors: cyclosporine A and tacrolimus. These drugs commonly constitute the basis of treatment of transplant recipients, since immunosuppressive therapy is mostly based on three rather than one drug. Cyclosporine A is one of the primary immunosuppressive drugs [40,41]. Biochemically, it is a cyclic peptide with eleven amino acids, and it is a metabolic peptide of the fungus *Tolypocladium inflatum*. The drug works by inhibiting calcineurin. The formation of a complex with immunophilin, called cyclophilin, occurs, preventing the translocation of activated nuclear T-cell transcription factor (NF-AT). It also inhibits the activation of other transcription factors, including NF-κB, preventing the induction of genes encoding various cytokines, particularly IL-2 [42]. The metabolism of CsA occurs mainly through the cytochrome P (CYP) 450A3 enzyme system and, to a lesser extent, in the gastrointestinal tract and kidneys to metabolites, all of which are much less active than the original compound.

Tacrolimus seems to be one hundred times stronger than CsA [25]. This drug inhibits cellular activity and the humoral immune response by various mechanisms with the main effect on calcineurin inhibition [43]. This inhibition occurs through the formation of a complex with the immunophilin FK 506, preventing NF-AT translocation, which promotes T helper cell proliferation mediated by IL-2 [25]. Furthermore, tacrolimus crosses the human placenta, and concentrations in breast milk are related to plasma concentrations. The drug undergoes extensive metabolism in the liver, and less than 1% of the drug is excreted unchanged in the urine. To a much lesser extent, metabolism also occurs in the intestinal mucosa with metabolism mediated by the activity of CYP3A4 [25]. Clinically important aspects of tacrolimus use are toxicity and significant differences in pharmacokinetics and pharmacodynamics among individuals. The bioavailability of tacrolimus in individual patients ranges from 5 to 90%. For this reason, it is difficult to predict the optimal starting dose, adjust the maintenance treatment regimen and monitor the risk of adverse effects or treatment failure [44]. The most significant side effects are acute and chronic nephrotoxicity, hypertension, neurotoxicity, diabetes, hirsutism, cholestasis, hyperuricemia, and gingival hyperplasia [41,42,45].

Calcineurine inhibitors cause multi-systemic disturbances, including the urinary system, gastrointestinal tract, and circulatory system. Wilk et al. [46] observed that regimens based on CNIs lead to hepatocyte apoptosis. What is more, the tacrolimus-based protocol was the most hepatotoxic immunosuppressive regiment, as evidenced by the highest percentage of apoptosis among all examined livers [46]. CNIs affect changes regarding immunity. Kabat-Koperska et al. [47] indicated qualitative, quantitative, and morphological changes in the immune system of infant rats born to pharmacologically immunosuppressed females. The thymus structure, spleen composition, and splenocyte IL-17 production were mostly affected in a drug regimen-dependent manner [47]. It should be also emphasised that CNIs disturb oxidative stress. Results presented by Wilk et al. [48] showed that only the group of rats treated with tacrolimus, mycophenolate mofetil, and prednisone indicated an increase in lipid peroxide concentration compared to the control group, although the difference was not statistically significant. A comparison of lipid peroxide concentration between the other treatment combinations and the control group showed a significant decrease. Additionally, a difference in lipid peroxide concentrations in the livers was observed between the cyclosporine A group and tacrolimus group [48].

CNIs lead to changes in aspect of the proteinogram of plasma proteins. Kędzierska et al. [49] observed that in the groups of rats treated with regimens based on CyA, a higher KIM-1 concentration was found and KIM-1 was positively correlated to 170 kDa protein (alpha2-macroglobulin). Ha and Mun [50] described the side effects of CNIs regarding metalloproteinases associated with the circulatory system. The authors suggest adverse effects of CsA on the vessel structure by the violation of balance of the specific endopeptidases; there was an activity increase in MMP-1, MMP-3, MMP-8, MMP-9, and MMP-13, and there was an activity decrease in MMP-2 [50]. Additionally, arteries of animals that were treated with cyclosporine indicated spot thickening of the endothelium connected with the increased depositing of extracellular matrix components, which led to narrowed vessel lumen [51]. Surówka et al. [52] examined rats aortas treated with different immunosuppressive drugs. The most profound alterations regarding vessels were observed in calcineurin inhibitor-based protocols. Regimens based on tacrolimus, TMG protocol treatment, were characterised by the most numerous alterations, such as morphological changes. These changes included the thickness of the tunic media, wider distances between elastic lamellae, an increase in the number of vascular smooth muscle cells, and changes in collagen deposition. The authors suggested that this observation was associated with MMP-2, MMP-9/TIMP-2, and TIMP-1 imbalances, which was also determined and noticed [52].

### 3.3. Mammalian Target of Rapamycin Inhibitors

Sirolimus and everolimus, which belong to the mammalian target of rapamycin inhibitors (mTORi), have an identical mechanism of action. These drugs inhibit T- and B-cell proliferation and differentiation, antibody production, and the proliferation of non-immune cells (fibroblasts, endothelial cells, hepatocytes, and smooth muscle cells) [53,54]. These drugs are rapidly absorbed after oral administration and, when taken with food, their bioavailability is affected. Other factors that affect bioavailability include hepatic metabolism by CYP3A4, intestinal glycoprotein countertransport, intestinal membrane permeability, and hepatic first-pass metabolism. Its mechanism is unknown, but it is likely a cellular autoimmune response following exposure to latent antigens or late hypersensitivity mediated by T lymphocytes. Its occurrence determines the discontinuation of mTORs and a change in the immunosuppressive treatment regimen. It is noticed that protocols based on CNI and mTORs exhibit protective properties regarding graft function [55].

The most common side effects of mTOR inhibitors are pneumonitis, thrombotic microangiopathy, surgical scar infection or late healing, lymphocele, productive surgical drainage, post-transplant diabetes mellitus, hypertriglyceridaemia, hypercgolesterolaemia, proteinuria, and oedemas [55,56].

Kim et al. [57] indicated increased incidence of neuropsychiatric disease in kidney transplant patients treated with rapamycin through the suppression of neural stem cells. Rapamycin also affects the oral cavity. An et al. [58] demonstrate that a short-term treatment with rapamycin in aged mice improved the condition of the oral cavity regarding periodontal bone loss, periodontal inflammation, and pathogenic changes to the oral microbiome [58].

### 3.4. Antiproliferatives

One of the drugs that belongs to antiproliferatives is azathioprine, which is a purine analogue that mediates its effects by preventing the synthesis of nucleic acids. The drug inhibits the activation, differentiation, and proliferation of lymphocytes and reduces the activity of NK cells. These mechanisms produce an immunosuppressive effect, preventing the proliferation of cells involved in the initiation and enhancement of the immune response. The active compound is thiosinic acid, which is a purine analogue of guanine that interferes with RNA and DNA synthesis, resulting in cytotoxic effects on leukocytes [1,25,59].

In addition to azathioprine, mycophenolic acid (MPA) and its two clinically used derivatives, mycophenolate sodium and mycophenolate mofetil, are among the most widely used immunosuppressive drugs that inhibit lymphocyte proliferation in preventing the rejection of the transplanted kidney [1,25]. MPA is a reversible and incompetent inhibitor of the enzyme inosinomonophosphate dehydrogenase (IMPDH). Inhibition of the enzyme’s activity results in impaired de novo guanine synthesis and DNA replication. This blocks the proliferation of T and B lymphocytes, which are dependent on the de novo purine synthesis pathway. MPAs also inhibit glycoprotein production by lymphocytes and monocytes, reducing their adhesion to endothelial cells [60].

Regarding the effects of mycophenolates, Yung et al. [61] examined MMF and cyclophosphamid in lupus nephritis and indicated that MMF seems to have more rapid and more sustained control of inflammatory and fibrotic disease processes regarding renal tissue than cyclophosphamid [61]. They suggest that MMF used with glucocorticosteroids decreased fibronectin expression in the kidneys of mice [61]. It should be emphasised that besides side effects regarding renal tissue, immunosuppressive drugs may lead to multi-systemic disorders. Schwarze et al. [62] examined mycophenolate mofetil on cardiac allograft survival and cardiac allograft vasculopathy in miniature swine. It turned out that mycophenolate mofetil resulted in longer allograft survival than a similar course of cyclosporine. Moreover, mycophenolate mofetil reduced the prevalence of cardiac allograft vasculopathy compared with cyclosporine-treated animals. The authors explained that the salutary effect of mycophenolate mofetil may be associated with its ability to decrease interferon-γ expression in the myocardium and prevent the generation of alloantibodies [62]. Additionally, Rathinam et al. [63] described the influence of mycophenolate mofetil on uveitis [63]. It turned out that among adults with non-infectious uveitis, the use of mycophenolate mofetil did not exhibit better properties regarding inflammation than methotrexate [63].

### 3.5. Glucocorticosteroids

Glucocorticosteroids are drugs commonly used in patients suffering from diseases with various aetiologies, including autoaggressive diseases and dermatological disorders [64]. Glucosteroids constitute a major component of immunosuppressive therapy to prevent organ rejection. Recently, patients with stabile function of the graft do not need to use them [65]. There are two glucocorticosteroids used in renal transplant recipients: methylprednisolone and prednisone [25,66]. These drugs exhibit anti-inflammatory and immunomodulatory effects in addition to their immunosuppressive effects [65].

Wang et al. [67] suggest that glycyrrihizic acid and prednisone improve renal function, relieve the renal morphological changes, and decrease interstitial fibrosis in rats [67]. However, some researchers, based on the examination of immunosuppressive regimens, where prednisone was included, indicated the severe effect of these drugs on various tissues, including kidney [46,47,52]. Nevertheless, these studies concerned triple-drug protocols; therefore, the role of prednisone on organs was not clear [46,47,52]. Interestingly, treatment with daily prednisone compared with intermittent prednisone (alternating 10 days on and 10 days off) led to a significant improvement over 3 years in a composite outcome comprising measures of motor function, pulmonary function, and general satisfaction among patients with Duchenne muscular dystrophy [68].

Of note, immunosuppressive protocols have been developed based on an appropriate combination of drugs with different mechanisms of action. It should be added that immunosuppressive treatment needs to be personalised, and hospitals and clinics have differing protocols and preparations depending on experience. Established protocols may need to be modified in situations involving donor, organ, or recipient characteristics. The usual variables considered are donor type (living or deceased), HLA matching, organ quality, donor age, recipient immune risk, comorbidities, and possible infections. These variables can influence the choice of pharmacological agents and the decision to use induction with antibodies. Current protocols are usually based on triple-drug therapy containing calcineurin inhibitors, corticosteroids, and antiproliferative drugs. In addition, mono- or polyclonal antibodies are also used in induction therapy [28]. It is important to note that the intensity of immunosuppression is determined by immunologic risk, and because of this, patients are classified according to variables related to immunologic risk. The first category consists of patients with low immunologic risk. Individuals in this group are recipients from HLA-identical donors and elderly patients. Antibody induction may not be necessary, and pharmacological immunosuppression is usually carried out with corticosteroids, calcineurin inhibitors, and mycophenolate or azathioprine. In these patients, it is also possible to use protocols that do not require continuous steroid use, and in that case, induction with antibodies is recommended [60].

## 4. Conversions

**Figure 6:**
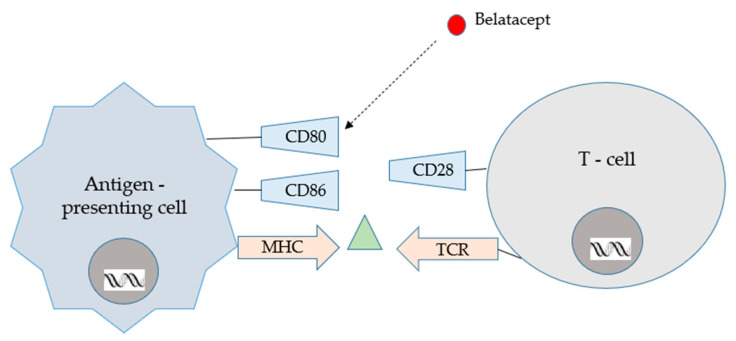
Belatacept mode of action [62].

Conversion from CNIs to belatacept seems to be a positive, new alternative for organ recipients. CNIs, when long used, may cause many side effects; however, one of the most harmful is neurotoxicity. Recent studies indicate that conversion from a CNI- to a belatacept-based regimen in stable renal graft recipients 6–60 months after transplantation seems to be well tolerated and associated with similar patient and graft survival rates. According to Budde et al. [69], graft function was improved with belatacept conversion and increased over time compared with CNI continuation. Co-stimulation blockade with belatacept increased BPAR rate, yet lower de novo DSAs were reported with belatacept conversion compared with CNI continuation. It should be emphasised that conversion from CNI to belatacept may be an important alternative treatment for renal allograft recipients [69]. Belatacept’s mode of action is presented in Figure 6.

It is important that conversion to belatacept be avoided when the patient is EBV positive, since patients converted to belatacept indicated a significant risk of opportunistic infections [70,71,72].

Recently, conversion from CNI-based to belatacept-based immunosuppression has been commonly used in many transplant centres; however, numerous protocols have emerged in lieu of a standardised protocol. Yazdi et al. [73] have described belatacept conversion protocols and outcomes in renal transplant recipients. Protocols were defined by the initial dose, induction regimen, and CNI taper. Rejection rates were low and may be influenced by exposure to maintenance immunosuppression during and after conversion. Most patients showed stabilisation and improvement in creatinine post-conversion, with the largest effect in those with an early conversion and serum creatinine > 2.0 mg/dL. A large variety of belatacept conversion protocols are being used and vary by initial belatacept dose, induction frequency, and calcineurin inhibitor (CNI) tapering scheme. Moreover, renal function improved significantly in those without post-conversion rejection. On the contrary, those with rejection did not experience an improvement in renal function. The most robust improvement in kidney function was seen in those converted within 1 year of transplant with a creatinine ≥ 2.0 mg/dL at the time of conversion. Furthermore, according to Yazdi et al. [73], lower tacrolimus exposure at the time of conversion and lower mycophenolic acid dose tended to be associated with rejection. Attention should be paid to the CNI tapering regimen, CNI exposure, and maintenance mycophenolic acid dosing during conversion to prevent rejection.

Regarding BENEFIT trials, there are fewer patients with de novo donor-specific antibody (DSA) in the belatacept arm [67,68]. Studies on mouse model have revealed that the administration of abatacept (CTLA4-Ig) decreased de novo and memory alloantibody responses [74,75,76]. Furthermore, an association was found with belatacept-based immunosuppression and reduced the conversion of IgM DSA to the more deleterious IgG DSA [77]. A significantly increased rate of rejection was noted in HLA-sensitised patients switched within the first year following transplant. Yazdi et al. [73] described data where rejection categorised as borderline in patients with DSA presented at the time of conversion, and there was no rejection among early conversions in the DSA-positive population.

Another way to improve graft function and avoid its rejection while minimising other side effects is conversion from CNIs to mTORs. Data regarding the clinical advantage of this conversion strategy remain conflicting. Budde et al. [78] showed that mTORi combined with CNI withdrawal improved kidney function while maintaining efficacy and safety. However, many researchers reported that regimens based on mTORs not only failed to show any overall clinical benefit, but also mTORs conversion led to many adverse events [79] and discontinuations [80]. Undoubtedly, further evidence for the validity of mTORi conversion strategy is urgently needed. According to Zeng et al. [81], post-transplant patients have better graft function and lower incidence of malignancy after conversion from CNI to mTORi therapy. However, this conversion strategy may be prevented by the higher drug discontinuation rate due to mTORs’ associated side effects, such as infection, proteinuria, leukopenia, acne, and mouth ulcer, indicating that conversion therapy may only be a treatment option in selected patients. It should be added that conversion should not be used in the first 6–8 weeks after transplantation to avoid wound-healing disruption.

## 5. Complications Associated with Usage of Immunosuppressive Drugs

Immunosuppressive drugs are essential for transplant function, but as mentioned above, they can lead to a wide variety of multi-organ disorders and diseases that cause mortality, including cardiovascular disorders. It is worth noting that post-transplant diabetes, hypertension and hyperlipidaemia are among the significant complications caused by immunosuppressive drugs. Therefore, it is advisable to limit, if possible, the use of glucocorticoids. In addition, the prevention and treatment of anaemia is also important. In this case, vitamin D supplementation, treatment with folic acid, vitamin B6 and B12, and the use of anti-inflammatory and antioxidant drugs can be included in the treatment of kidney transplant recipients. In addition, a healthy lifestyle, including smoking cessation, a healthy diet and, most importantly, physical activity are key factors in reducing cardiovascular risk [82].

Cancer is the second most common cause of death in kidney transplant patients. These cancers mainly include renal cell carcinoma and skin cancer. Compared to the general population, kidney transplant recipients belong to a group of patients with a higher risk of developing renal cell carcinoma. About ninety percent of renal cell carcinomas are believed to develop in healthy kidneys. However, the incidence of renal cell carcinoma in a transplanted kidney is rare and is estimated to be around 0.1% [83]. Skin cancer is the most common and aggressive type of cancer in kidney transplant recipients. The most frequently reported skin cancers in the aforementioned group of patients include cutaneous squamous cell carcinoma, basal cell carcinoma, Kaposi’s sarcoma and malignant melanoma [83]. Although Kaposi’s sarcoma is a rare cancer, its incidence in transplant recipients is more than 100 times higher than in the general population [83]. It is worth noting that kidney transplant recipients treated with calcineurin inhibitors are at exceptional risk for Kaposi’s sarcoma. As for skin cancer, melanoma is also a frequently reported cancer. If the aforementioned cancer is diagnosed, the conversion of a CsA or Tac-based immunosuppressive regimen to an mTOR inhibitor seems to be the optimal solution with apparent positive effects. As mentioned in this review, the choice of immunosuppressive treatment is crucial and should be individualized for each patient based on disease aetiology and graft function.

Another complication associated with the use of immunosuppressive drugs is viral infections [84]. Epstein–Barr virus (EBV) is a human herpesvirus infecting approximately 90% of adults [84]. The aforementioned virus can manifest asymptomatic viremia, infectious mononucleosis syndrome or involvement of other organs, such as hepatitis, myocarditis and pancreatitis. The most symptomatic infections in kidney transplant recipients are primary infections, which are probably related to reactivation of the donor virus. The most worrisome manifestation of EBV is post-transplant lymphoproliferative disease [84]. In addition, cytomegalovirus (CMV) is a common virus in kidney transplant recipients. The risk of CMV infection depends primarily on the CMV serological status of the donor and recipient. As for immunosuppressive drugs, belatacept is associated with an increased risk of primary CMV infection and a prolonged course of viral replication in patients at high risk of CMV infection [84]. Moreover, BK polyomavirus and norovirus are also complications that occur after organ transplantation [84]. Primary infection with BK polyomavirus occurs in childhood, and 80–90% of adults are exposed to it. The virus remains latent in the renal tubules and uroepithelium, while norovirus in immunocompromised patients can develop chronic norovirus infections associated with recurrent and recurring episodes of watery diarrhoea that can last from months to years [84].

Due to the properties of immunosuppression, which transplant recipients must constantly apply, the aforementioned group of patients is extremely vulnerable to bacterial infections. Urinary tract infections of various aetiologies are the most common infection in kidney transplant recipients. They occur most frequently in the first year after transplantation, and the incidence ranges from 7% to 80% [84]. Gram-negative bacteria cause up to 90% of cases, and Escherichia coli was the most commonly reported. Infection with C. difficile is five times more likely in hospitalised solid organ transplant recipients compared to the general population [84].

Invasive fungal infections pose a serious threat to kidney transplant recipients. Fungal infections in developing countries have a significant impact on patient and graft survival. Kidney transplant recipients are at risk for fungal infections mainly in the early period after surgery. The most common fungal infections are caused by fungi of the Candida genus and can be replaced by more benign infections with endemic mycoses such as mucormycosis, histoplasmosis, blastomycosis and coccidioidomycosis [84,85].

## 6. Other Options of Immunosuppressive Treatment Used by Renal Transplant Recipients

Of note, besides drugs that conclude the maintenance of multiple schemes of treatment used in renal transplant recipients, there are many different medicines with immunosuppressive properties. These drugs constitute an alternative medical basis for organ recipients; however, they are mainly used in a wide spectrum of diseases, including cancers, rheumatoid arthritis, psoriatic arthritis, ulcerative colitis, and/or widely in dermatological disease.

In addition to plasma exchange and intravenous immunoglobulin, a number of other add-on therapies have been tried in small trials with no consistent benefit, including anti-CD20, proteasome inhibitors, complement inhibitors, interleukin-6 receptor blockers, and the immunoglobulin G-degrading enzyme Streptococcus pyogenes (called IdeS) [86]. IdeS could be a ground-breaking new method of desensitising patients who might otherwise have no hope of receiving a life-saving transplant [87].

Another immunosuppressive drug used in renal transplant recipients is tofacitinib [31], which is a potent selective and reversible inhibitor of the Janus kinases JAK1, JAK2, and JAK3, as well as TyK2 (a non-receptor tyrosine-protein kinase). These enzymes affect immune cell function through the phosphorylation and activation of STAT proteins. Blockade of the formation of the JAK–STAT complex leads to a reduction in the immune response and a decrease in the pro-inflammatory effects of cytokines such as IL-2, IL-4, IL-7, IL-15, IL-21, IL-6, IL-9, and IFN-alpha and IFN-beta [31]. Tofacitinib is also followed by a significant reduction in serum C-reactive protein (CRP) levels. It has also been studied in the context of immunosuppressive effects in the prevention of kidney transplant rejection, but studies have shown no significant differences between tofacitinib and cyclosporine-A in terms of prevention of acute biopsy. Patient and graft survival was also similar [31,88].

Furthermore, bortezomib constitutes another option in the treatment of organ recipients [31]. It has been revealed that this drug, if administered prior to transplant or soon thereafter, decreases donor-specific antibody levels and improves transplanted organ survival. Bortezomid as an option for antibody-mediated rejection after transplant remains unclear, with limited evidence supporting its long-term success [89]. Bortezomid is treated as an alternative for renal transplant recipients, since it has effects on circulating B cells and TH cells. It can lead to blockade of the T-cell cycle, which leads to the apoptosis of TH cells, and causes an IL-6 reduction [31,90]. Of note, bortezomid is mainly used as anti-cancer drug against haematological disorders [91]. It can be used as a substitute for organ recipients; however, it may lead to peripheral sensory neuropathy, nausea, diarrhoea, thrombocytopenia, neutropenia, and fatigue [92]. Bortezomib seems to be a promising early desensitising medicine in transplantology, and high short-term success rates have been observed.

However, like in every newly used drug and method, more research and clinical trials would be useful. Its effectiveness and optimal administration time in relation to transplant surgery should be also expanded, and more data are needed.

## 7. Future Possibilities

Transplantation is a rapidly developing field of medicine, but there are still many problematic and unclear issues. An extremely important aspect seems to be the development of immunosuppressive drugs that would not interfere with the function of the organ through toxic effects and would ensure the proper functioning of the transplanted organ. Therapeutic drug monitoring (TDM) is one of the first precision medicine tools, as it aims to individualize drug dosage based on patient characteristics (pharmacogenetics, demographics, clinical information, etc.) or by measuring drug concentrations in the blood [93]. Monitoring drug concentrations is crucial for kidney transplant recipients, as it is important to choose the best minimum dose of said drugs while avoiding side effects. Cyclosporine in excessive doses leads to irreversible kidney damage [94]. Monitoring the area under the concentration–time curve (AUC) of CsA has evolved, but recently, it has been recommended to monitor CsA blood concentrations 12 h after administration. The highest concentration of this drug is reached 6 h after administration. Prospective studies are currently underway to compare pre-dose concentration monitoring with infrequent or limited AUC monitoring [94]. For tacrolimus, whole blood concentration measurements are used for monitoring. The pharmacokinetics of tacrolimus is highly variable. However, tacrolimus concentration monitoring is similar to CsA [94]. Monitoring mycophenolate mofetil with 6 h sampling is associated with a high failure rate due to delayed absorption. There are patients who may require a full 12 h AUC to effectively capture mycophenolic acid exposure. For patients who require the therapeutic monitoring of MPA, MMF is currently the most practical therapeutic option, and mycophenolate sodium administered as enteral tablets may be preferred for use in those renal transplant recipients who do not require therapeutic monitoring of the drug [95]. Weekly monitoring of sirolimus blood levels is recommended for the first month and every two weeks for the following month. Further monitoring is recommended only when clinically warranted [96].

It is worth noting that monitoring immunosuppressant drugs using a home-based method would be promising and helpful for organ recipients, as consistent drug levels are extremely important. Zwart et al. [97] described a home-based dry blood drop method for monitoring tacrolimus and mycophenolic acid dosing after kidney transplantation. Home dry blood drop (DBS) sampling has the potential to replace conventional TDM sampling in the clinic [93,97]. In addition, a helpful option for organ transplant recipients would be therapeutic point-of-care drug monitoring for the precise dosing of immunosuppressive drugs, as described by Taddeo et al. Among others, point-of-care-based monitoring could improve care for transplant recipients by reducing waiting times by allowing regular measurements. Regular monitoring is key to obtaining pharmacokinetic data, responding more quickly to problems and increasing patient comfort [93].

Monitoring recipient immunity based on viral status appears to be an equally effective method for successful transplantation. A new tool may be monitoring Torque-teno virus (TTV) titres. TTV levels have been shown to be associated with the potency of immunosuppressive drugs. However, further research is needed to evaluate TTV measurement as a tool to monitor immunosuppressive drugs for difficult clinical outcomes, such as the presence of donor-specific antibodies, rejection or infection [98,99].

Another important transplant-related factor that may be of fundamental importance in the future is the monitoring of free donor-derived DNA (ddcfDNA), which is a non-invasive indicator of allograft damage [100]. Interestingly, elevated levels of ddcfDNA occur in cases of rejection, infection and acute tubular necrosis. The optimal methodology for determining ddcfDNA, the optimal threshold for no damage, and what level is most useful (absolute amount or fraction of total cfDNA) remain to be determined. According to Oellerich et al. [100], post-transplantation ddcfDNA determinations are useful to avoid unnecessary biopsies induced by rising plasma creatinine levels and to detect asymptomatic subclinical graft damage, including that caused by inadequate immunosuppression. It should be noted that more studies are needed, particularly randomized trials and cost-effectiveness analyses, and no firm recommendations can be made at present [100].

In addition, the Molecular Microscopic Diagnostic System (MMDS), a microarray-based system that assesses gene expression in renal allograft biopsies, appears to be important in predicting T cell or antibody-mediated rejection. Importantly, this method can be effective even before lesions are visible by standard pathological methods. According to Argani [101], MMDS is an alternative, novel method in addition to the histology-based approach and allows risk stratification that can guide clinical management and clinical trials in transplantation [101].

As more and more patients suffer from kidney disease, xenotransplantation may be the solution. However, the topic seems controversial, and more data are needed to expand knowledge in this area. Advances in genetic engineering, immunosuppression and tolerance have improved the survival rate of porcine organs in primates. In 2022, a pig heart was transplanted into a patient with heart failure and functioned for seven weeks, and xenotransplantation experiments using pig kidneys in deceased human recipients have provided encouraging data [102]. Sykes et al. [102] suggest that methods of mixed hematopoietic chimerism and thymus transplantation have been successfully used to tolerate human T lymphocytes, B lymphocytes and NK cells in mice with human immune systems. Advances in the application of tolerance methods in NHPs, including genetic engineering of source pigs, support their potential to provide a long-term solution to the powerful immune barriers to xenotransplantation [102].

## 8. Conclusions

There are many immunosuppressive drugs, and they are used in many combinations; however, the lifespan/duration of transplanted organ function, including renal grafts, is still unsatisfactory. Today, the search for new immunosuppressive drugs continues, with the aim to minimise the side effects of these drugs and simultaneously prevent graft rejection. The aim of current research is to develop new immunosuppressive drugs that will effectively prevent the process of transplant organ rejection, while interfering as little as possible with the patient’s immune system, and hence give rise to severe side effects. Prolonging patient and graft survival and improving the function of the transplanted kidney are the most important challenges in modern transplantology.

## Data Availability

Not applicable.
